# Performance characteristics of prostate-specific antigen density and biopsy core details to predict oncological outcome in patients with intermediate to high-risk prostate cancer underwent robot-assisted radical prostatectomy

**DOI:** 10.1186/s12894-017-0238-y

**Published:** 2017-06-23

**Authors:** Masahiro Yashi, Akinori Nukui, Yuumi Tokura, Kohei Takei, Issei Suzuki, Kazumasa Sakamoto, Hideo Yuki, Tsunehito Kambara, Hironori Betsunoh, Hideyuki Abe, Yoshitatsu Fukabori, Yoshimasa Nakazato, Yasushi Kaji, Takao Kamai

**Affiliations:** 1 Department of Urology, Dokkyo Medical University, 880 Kitakobayashi, Mibu, Shimotsuga, Tochigi 321-0293 Japan; 2 Department of Pathology, Dokkyo Medical University, Tochigi, Japan; 3 Department of Radiology, Dokkyo Medical University, Tochigi, Japan

**Keywords:** Predictive factor, Performance characteristics, Prostate-specific antigen density, Biopsy core details, Robot-assisted radical prostatectomy

## Abstract

**Background:**

Many urologic surgeons refer to biopsy core details for decision making in cases of localized prostate cancer (PCa) to determine whether an extended resection and/or lymph node dissection should be performed. Furthermore, recent reports emphasize the predictive value of prostate-specific antigen density (PSAD) for further risk stratification, not only for low-risk PCa, but also for intermediate- and high-risk PCa. This study focused on these parameters and compared respective predictive impact on oncologic outcomes in Japanese PCa patients.

**Methods:**

Two-hundred and fifty patients with intermediate- and high-risk PCa according to the National Comprehensive Cancer Network (NCCN) classification, that underwent robot-assisted radical prostatectomy at a single institution, and with observation periods of longer than 6 months were enrolled. None of the patients received hormonal treatments including antiandrogens, luteinizing hormone-releasing hormone analogues, or 5-alpha reductase inhibitors preoperatively. PSAD and biopsy core details, including the percentage of positive cores and the maximum percentage of cancer extent in each positive core, were analyzed in association with unfavorable pathologic results of prostatectomy specimens, and further with biochemical recurrence. The cut-off values of potential predictive factors were set through receiver-operating characteristic curve analyses.

**Results:**

In the entire cohort, a higher PSAD, the percentage of positive cores, and maximum percentage of cancer extent in each positive core were independently associated with advanced tumor stage ≥ pT3 and an increased index tumor volume > 0.718 ml. NCCN classification showed an association with a tumor stage ≥ pT3 and a Gleason score ≥8, and the attribution of biochemical recurrence was also sustained. In each NCCN risk group, these preoperative factors showed various associations with unfavorable pathological results. In the intermediate-risk group, the percentage of positive cores showed an independent predictive value for biochemical recurrence. In the high-risk group, PSAD showed an independent predictive value.

**Conclusions:**

PSAD and biopsy core details have different performance characteristics for the prediction of oncologic outcomes in each NCCN risk group. Despite the need for further confirmation of the results with a larger cohort and longer observation, these factors are important as preoperative predictors in addition to the NCCN classification for a urologic surgeon to choose a surgical strategy.

**Electronic supplementary material:**

The online version of this article (doi:10.1186/s12894-017-0238-y) contains supplementary material, which is available to authorized users.

## Background

Prostate cancer (PCa) has recently become the most prevalent malignant disease and was categorized as the 6th highest cause of death in Japanese men [1]. In addition, radical prostatectomy is a first-line therapy, and a robot-assisted procedure has become a standard method for patients with localized PCa. Despite the widespread use of prostate-specific antigen (PSA) screening, a saturation biopsy protocol with multiple cores, and progress in diagnostic imaging, contemporary PCa patients still include a highly heterogeneous population of oncological outcomes. The established risk stratification system consisting of PSA, Gleason score (GS), and clinical T stage seem to insufficiently identify patients with unfavorable pathologic features preoperatively, leading to biochemical, local, and systemic recurrence [2]. We previously raised this issue from data that selected Japanese patients with low-risk PCa who still demonstrated advanced-stage (≥ pT3) disease at around 15% [3]. Another Japanese study group compared five established risk stratification systems in their cohort, and found that all stratification systems could not discriminate between low- and intermediate-risk groups in terms of biochemical recurrence-free rate [4]. In view of racial differences, criteria developed from a Western cohort analysis cannot always be applied to Japanese or Asian patients [5].

Detailed information obtained from prostate biopsy has been suggested to include predictors of oncological outcomes since the sextant biopsy era [6], and protocols of multiple core biopsy with greater than 12 cores has enhanced its predictive value [7]. Currently, many urologic surgeons refer to biopsy core details for further risk assessment and decision making, and to determine whether an extended resection and/or lymph node dissection should be performed during radical prostatectomy. Furthermore, pretreatment use of PSA density (PSAD) for further risk stratification has been emphasized, not only for patients with low-risk PCa, but also those with intermediate- and high-risk PCa [8].

This study focused on these preoperative parameters and compared predictive impact on oncological outcomes, including unfavorable pathologic features and biochemical recurrence in Japanese patients with intermediate- and high-risk PCa.

## Aims of study

The established risk stratification system is convenient but insufficient to make decisions for patients who need definitive therapy and for a urologic surgeon to decide upon the surgical strategy. On the other hand, prediction nomograms can offer individualized risk of PCa, but several variations exist and an ambiguity remains in the interpretation of the calculated probabilities. Accordingly, we must continue to refine the risk stratification system adapted to the demands of contemporary PCa patients. The aim of this study is to identify potential factors associated with oncological outcomes in the entire cohort and in each risk group, and to clarify the performance characteristics of predictive ability. Finally, we propose an improved risk assessment from the results.

## Methods

### Inclusion criteria of patients

The study population consisted of 250 consecutive patients that underwent robot-assisted radical prostatectomy between October 2012 and October 2016 at a single Japanese academic hospital with observation periods longer than 6 months. Of these patients, 155 patients were classified as being at intermediate-risk defined as having at least one characteristic among clinical stage T2b-c or GS of 7 or PSA level of 10 to 20 ng/mL, and 95 patients were classified as high-risk defined as having at least one characteristic among clinical stage ≥ T3a or GS ≥8 or PSA level ≥20 ng/mL, according to the National Comprehensive Cancer Network (NCCN) classification [9]. Fourteen patients (14.7%) with either very-high-risk PCa featured by ≥5 cores with a Gleason sum of 8 to10, or multiple NCCN high-risk features were not excluded, and were analyzed as a high-risk group in this study. None of the patients received hormonal treatments including antiandrogens, luteinizing hormone-releasing hormone analogues, or 5-alpha reductase inhibitors preoperatively. Robot-assisted radical prostatectomy was carried out using an intraperitoneal anterior approach by six surgeons. Lymph node invasion (LNI) risk was evaluated by using a Briganti nomogram [7], and lymph nodes were dissected in 70% of patients. Patients with a LNI risk ≥5% underwent extended dissection, and those with a LNI risk <5% underwent standard dissection or were spared dissection according to the surgeon’s decision.

### Preoperative clinical data, biopsy, and radiographic findings

We retrospectively reviewed the records of clinical data, findings of multiple core biopsy, and radiographic images. Preoperative patient characteristics are provided in Table 1. PSAD was determined as a pre-biopsy PSA value divided by magnetic resonance imaging (MRI)-estimated prostate volume [10]. Systematic prostate biopsy was performed through a transperineal approach without a MRI-fusion method at our hospital and related facilities. The median number of biopsy cores per procedure was 20 and no patient had fewer than 10 cores. Biopsy specimens were evaluated for GS, number of cores involved with cancer, maximum percentage of cancer extent in each positive core, and percentage of cancer measured by subtracting the intervening benign glands. Gleason scoring of the biopsy specimens was done according to the International Society of Urological Pathology (ISUP) Consensus 2005 [11]. The findings of multiparametric MRI were centrally read by one specialist of urologic radiology, whether there were typical suspicious lesions for malignancy or not, and we did not use a Prostate Imaging Reporting and Data System (PIRADS) preoperatively in this study [12]. We comprehensively determined clinical T stage together with findings from the digital rectal examination.

**Table 1:** Preoperative patient characteristics

	Number (%) or Median (IQR)		
	Intermediate-risk	High-risk	*p*-value
Number	155 (100)	95 (100)	
Age (year)	66 (62–69)	67 (63–71)	0.197
PSA (ng/ml)	6.4 (5.0–8.6)	7.8 (5.9–11.8)	**<0.001**
PSA density (ng/ml/cc)	0.184 (0.136–0.259)	0.221 (0.170–0.332)	**<0.001**
Number of biopsy core (n)	20 (14–20)	18 (14–20)	0.127
Number of positive core (n)	3 (2–5)	4 (2–6)	**0.006**
% of positive cores (%)	16.7 (9.3–27.3)	21.4 (14.0–35.0)	**0.001**
% of positive cores dominant side (%)	30.0 (14.3–42.9)	37.5 (20.0–57.1)	**0.001**
% of cancer extent (%)	42.7 (21.4–66.7)	50.0 (28.3–70.0)	0.055
Biopsy Gleason score			
5–7	155 (100)	15 (15.8)	**<0.001**
8–9	0 (0)	80 (84.2)	
DRE T stage			
cT1	118 (76.1)	60 (63.2)	**0.035**
cT2a-c	37 (23.9)	33 (35.6)	
cT3a-b	0 (0)	2 (1.2)	
MRI T stage			
NA	0 (0)	1 (1.1)	**<0.001**
cT1	51 (32.9)	22 (23.2)	
cT2a-c	104 (67.1)	58 (61.1)	
cT3a-b	0 (0)	14 (14.7)	

*IQR* interquartile range, *% cancer extent* maximum % of cancer extent in each positive core, *DRE* digital rectal examination, Bold indicates statistically significant

### Evaluation of prostatectomy specimens

Prostatectomy specimens obtained through robot-assisted surgery were processed according to the Stanford protocol [13], were step sectioned transversely at 4 mm intervals, and mounted as half or quarter sections for microscopic evaluation. These were evaluated for GS, extraprostatic extension, surgical margin status, seminal vesicle invasion, and tumor volume. Gleason scoring was also done as recommended in the ISUP Consensus 2005 [11]. One specialist of urologic pathology centrally evaluated the histopathology. Prostate cancer volume was estimated from the three-dimensional measurements of the index tumor, using an ellipsoid formula without correction by a shrinkage factor due to formalin fixation [14]: major diameter × minor diameter × anteroposterior diameter × Pi/6. The anteroposterior diameter was estimated from the number of step sections of 4 mm occupied by cancer.

### Statistical analyses

PSAD and biopsy core details, including the percentage of positive cores, the percentage of positive cores from the dominant side, and the maximum percentage of cancer extent in each positive core were analyzed in association with unfavorable pathologic results of prostatectomy specimens, and further with biochemical recurrence. In this study, biochemical recurrence was defined as a PSA level greater than 0.1 ng/mL with subsequent rising PSA. When the PSA level did not decline to less than 0.1 ng/mL after prostatectomy, the date of surgery was defined as that of recurrence (immediate recurrence).

Quantitative data were compared using a Mann–Whitney *U* test, and qualitative data were compared using a Fisher’s exact test. The cut-off values of PSAD, percentage of positive cores, percentage of positive cores from the dominant side, and maximum percentage of cancer extent in each positive core were set to be 0.345 ng/ml/cc, 21.4%, 37.5%, and 55.6%, respectively for the entire cohort; 0.190 ng/ml/cc, 21.4%, 36.4%, and 57.1% respectively for the intermediate-risk group; and 0.345 ng/ml/cc, 35.0%, 40%, and 55.6% respectively for the high-risk group by using receiver-operating characteristic curve analyses for deciding the effective point of judging biochemical recurrence.

Logistic regression and Cox proportional hazards regression models were used for univariate and multivariate analyses, and a stepwise selection procedure was used to elucidate significant factors. Recurrence-free survival was estimated using the Kaplan–Meier method, and differences were compared with the log-rank test. All statistical analyses were performed with EZR, which is a graphical user interface for R (The R Foundation for Statistical Computing, version 2.13.0). All statistical tests were two-sided, with *p*-values of less than 0.05 considered to be statistically significant.

## Results

In the preoperative characteristics indicated in Table 1, significant differences were observed in PSA (6.4 versus 7.8 ng/ml in median value, *p* < 0.001), PSAD (0.184 versus 0.221 ng/ml/cc in median value, *p* < 0.001), the number of positive cores (3 versus 4 in median value, *p* = 0.006), the percentage of positive cores (16.7 versus 21.4% in median value, *p* = 0.001), the percentage of positive cores from the dominant side (30.0 versus 37.5% in median value, *p* = 0.001), and the composition of biopsy GS, T stage by digital rectal examination or MRI (*p* < 0.001, 0.035, <0.001, respectively) between the intermediate- and high-risk groups, but not in the number of biopsy core (20 versus 18 in median value, *p* = 0.127), and the maximum percentage of cancer extent in each positive core (42.7 versus 50.0% in median value, *p* = 0.055). The tumor characteristics of the prostatectomy specimens are provided in Table 2. Significant differences were observed in the pathologic tumor stage (≥pT3: 20.0 versus 40.0%, *p* = 0.010), surgical margin status (positive: 12.3 versus 23.2%, *p* = 0.034), and prostatectomy GS (≥8: 11.6 versus 43.2%, *p* < 0.001) between the intermediate- and high-risk group, but not in index tumor volume (1.18 versus 1.05 cc in median value, *p* = 0.776).

**Table 2:** Tumor characteristics of prostatectomy specimens

	Number (%) or Median (IQR)		
	Intermediate-risk	High-risk	*p*-value
Number	155 (100)	95 (100)	
Pathologic T stage			
pT0	1 (0.6)	2 (2.1)	**0.010**
pT2a-c	123 (79.4)	55 (57.9)	
pT3a-b	31 (20.0)	36 (37.9)	
pT4	0 (0)	2 (2.1)	
Surgical margin			
Negative	136 (87.7)	73 (76.8)	**0.034**
Positive	19 (12.3)	22 (23.2)	
Prostatectomy Gleason score			
≤ 6	4 (2.6)	1 (1.1)	**<0.001**
7	133 (85.8)	53 (55.8)	
≥ 8	17 (11.0)	39 (41.1)	
NA	1 (0.6)	2 (2.1)	
Index tumor volume (cc)	1.18 (0.39–3.37)	1.05 (0.43–2.95)	0.776

*IQR* interquartile range, *NA* not available due to pT0, Bold indicates statistically significant

Among those unfavorable pathologic findings, advanced tumor stage ≥ pT3, increased index tumor volume >0.718 ml, and high GS ≥8 showed independent predictive value for biochemical recurrence, but surgical margin status did not show independent value in this study (see Additional file 1: Table S1). During a median follow-up of 24.5 months (interquartile range 14.0–36.0), it was discovered that 19 patients (12.3%) in the intermediate-risk group and 26 patients (27.4%) in the high-risk group developed biochemical recurrence.

The results of the univariate and multivariate analyses for the associations between preoperative factors and unfavorable pathologic results or biochemical recurrence are provided in Tables 3, 4, 5 and 6. In the entire cohort, a higher PSAD, percentage of positive cores, and maximum percentage of cancer extent in each positive core were independently associated with advanced tumor stage ≥ pT3 (odds ratio 4.370, 2.100, and 1.960, respectively) and increased index tumor volume >0.718 ml (odds ratio 2.860, 5.110, and 2.370, respectively). The higher percentage of positive cores from the dominant side showed independent association only with biochemical recurrence (odds ratio 2.648). NCCN classification showed an association with a tumor stage ≥ pT3 (odds ratio 1.910) and GS ≥8 (odds ratio 5.650), and the attribution of biochemical recurrence was also sustained (odds ratio 2.069). In each risk group of the NCCN classification, these preoperative factors showed various associations with unfavorable pathological results except for a high GS. In the intermediate-risk group, the higher percentage of positive cores and maximum percentage of cancer extent in each positive core showed a predictive value for biochemical recurrence in the univariate analysis, and a higher percentage of positive cores showed independent value in the multivariate analysis (odds ratio 3.910). In the high-risk group, PSAD and all biopsy core details showed predictive value in the univariate analysis, and a higher PSAD remained an independent value in the multivariate analysis (odds ratio 3.103). When analysis included the NCCN sub classification (very high-risk versus high-risk) in the high-risk group, the independent predictive value of PSAD remained unchanged, while the NCCN sub classification lost the independent value in the early stepwise selection procedure. Furthermore, PSAD showed more statistical superiority than PSA in the high-risk group.

**Table 3:** Factors associated with tumor stage ≥ pT3 in entire cohort and each risk group

	Entire cohort					
	Univariate analyses			Multivariate analyses		
	OR	95%CI	*p*-value	OR	95%CI	*p*-value
PSAD >0.345 vs ≤0.345 ng/ml/cc	5.850	2.820–12.20	**<0.001**	4.370	2.000–9.540	**<0.001**
% positive cores >21.4 vs ≤21.4%	3.150	1.770–5.630	**<0.001**	2.100	1.110–3.990	**0.028**
% dominant side >37.5 vs ≤37.5%	2.210	1.250–3.910	**0.006**			
% cancer extent >55.6 vs ≤55.6%	2.600	1.450–4.650	**0.001**	1.960	1.030–3.720	**0.041**
NCCN risk high vs intermediate	2.440	1.380–4.320	**0.002**	1.910	1.020–3.580	**0.042**
	Intermediate-risk					
	Univariate analyses			Multivariate analyses		
	OR	95%CI	*p*-value	OR	95%CI	*p*-value
PSAD >0.190 vs ≤0.190 ng/ml/cc	1.920	0.864–4.260	0.110			
% positive cores >21.4 vs ≤21.4%	3.260	1.450–7.320	**0.004**	3.260	1.450–7.320	**0.004**
% dominant side >36.4 vs ≤36.4%	2.400	1.070–5.380	**0.033**			
% cancer extent >57.1 vs ≤57.1%	2.180	0.970-4.900	0.059			
	High-risk					
	Univariate analyses			Multivariate analyses		
	OR	95%CI	*p*-value	OR	95%CI	*p*-value
PSAD >0.345 vs ≤0.345 ng/ml/cc	4.730	1.680–13.30	**0.003**	4.510	1.580–12.90	**0.004**
% positive cores >35.0 vs ≤35.0%	3.140	1.180–8.390	**0.023**			
% dominant side >40.0 vs ≤40.0%	1.880	0.811–4.360	0.141			
% cancer extent >55.6 vs ≤55.6%	2.810	1.170–6.760	**0.021**			

*OR* Odds ratio, *95%CI* 95% confidence interval, *% dominant side* % positive cores from dominant side, *% cancer extent* maximum % of cancer extent in each positive core, Bold indicates statistically significant

**Table 4:** Factors associated with index tumor volume > 0.718 in entire cohort and each risk group

	Entire cohort					
	Univariate analyses			Multivariate analyses		
	OR	95%CI	*p*-value	OR	95%CI	*p*-value
PSAD >0.345 vs ≤0.345 ng/ml/cc	3.780	1.520–9.440	**0.004**	2.860	1.060–7.680	**0.038**
% positive cores >21.4 vs ≤21.4%	6.640	3.520–12.50	**<0.001**	5.110	2.660–9.840	**<0.001**
% dominant side >37.5 vs ≤37.5%	6.140	3.250–11.60	**<0.001**			
% cancer extent >55.6 vs ≤55.6%	3.240	1.790–5.880	**<0.001**	2.370	1.250–4.520	**0.009**
NCCN risk high vs intermediate	1.220	0.717–2.070	0.465			
	Intermediate-risk					
	Univariate analyses			Multivariate analyses		
	OR	95%CI	*p*-value	OR	95%CI	*p*-value
PSAD >0.190 vs ≤0.190 ng/ml/cc	2.330	1.190–4.560	**0.014**			
% positive cores >21.4 vs ≤21.4%	8.050	3.320–19.50	**<0.001**	6.760	2.750–16.70	**<0.001**
% dominant side >36.4 vs ≤36.4%	5.900	2.610–13.40	**<0.001**			
% cancer extent >57.1 vs ≤57.1%	3.460	1.570–7.650	**0.002**	2.370	1.010–5.58	**0.048**
	High-risk					
	Univariate analyses			Multivariate analyses		
	OR	95%CI	*p*-value	OR	95%CI	*p*-value
PSAD >0.345 vs ≤0.345 ng/ml/cc	1.950	0.643–5.900	0.239			
% positive cores >35.0 vs ≤35.0%	7.380	1.610–33.90	**0.010**	7.380	1.610–33.90	**0.010**
% dominant side >40.0 vs ≤40.0%	3.560	1.390–9.100	**0.008**			
% cancer extent >55.6 vs ≤55.6%	2.410	0.959–6.050	0.061			

*OR* Odds ratio, *95%CI* 95% confidence interval, *% dominant side* % positive cores from dominant side, *% cancer extent* maximum % of cancer extent in each positive core, Bold indicates statistically significant

**Table 5:** Factors associated with pathological Gleason score ≥ 8 in entire cohort and each risk group

	Entire cohort		
	Univariate analyses		
	OR	95%CI	*p*-value
PSAD >0.345 vs ≤0.345 ng/ml/cc	1.510	0.697–3.280	0.296
% positive cores >21.4 vs ≤21.4%	1.350	0.739–2.450	0.331
% dominant side >37.5 vs ≤37.5%	1.440	0.788–2.620	0.237
% cancer extent >55.6 vs ≤55.6%	1.460	0.789–2.710	0.228
NCCN risk high vs intermediate	5.650	2.950–10.80	<**0.001**
	Intermediate-risk		
	Univariate analyses		
	OR	95%CI	*p*-value
PSAD >0.190 vs ≤0.190 ng/ml/cc	1.860	0.668–5.160	0.236
% positive cores >21.4 vs ≤21.4%	0.733	0.244–2.200	0.580
% dominant side >36.4 vs ≤36.4%	0.696	0.243–1.990	0.500
% cancer extent >57.1 vs ≤57.1%	1.660	0.590–4.650	0.338
	High-risk		
	Univariate analyses		
	OR	95%CI	*p*-value
PSAD >0.345 vs ≤0.345 ng/ml/cc	1.100	0.412-2.930	0.849
% positive cores >35.0 vs ≤35.0%	1.260	0.483–3.310	0.632
% dominant side >40.0 vs ≤40.0%	2.100	0.912–4.830	0.081
% cancer extent >55.6 vs ≤55.6%	1.020	0.437–2.390	0.959

*OR* Odds ratio, *95%CI* 95% confidence interval, *% dominant side* % positive cores from dominant side, *% cancer extent* maximum % of cancer extent in each positive core, Bold indicates statistically significant

**Table 6:** Factors associated with biochemical recurrence in entire cohort and each risk group

	Entire cohort					
	Univariate analyses			Multivariate analyses		
	HR	95%CI	*p*-value	HR	95%CI	*p*-value
PSAD >0.345 vs ≤0.345 ng/ml/	3.105	1.669–5.776	**<0.001**			
% positive cores >21.4 vs ≤21.4%	2.490	1.363–4.551	**0.003**			
% dominant side >37.5 vs ≤37.5%	3.793	2.016–7.136	**<0.001**	2.648	1.369–5.121	**0.004**
% cancer extent >55.6 vs ≤55.6%	3.301	1.778–6.130	**<0.001**	2.505	1.324–4.741	**0.005**
NCCN risk high vs intermediate	2.517	1.391–4.555	**0.002**	2.069	1.129–3.791	**0.019**
	Intermediate-risk					
	Univariate analyses			Multivariate analyses		
	HR	95%CI	*p*-value	HR	95%CI	*p*-value
PSAD >0.190 vs ≤0.190 ng/ml/cc	2.040	0.803–5.185	0.134			
% positive cores >21.4 vs ≤21.4%	3.910	1.485–10.29	**0.006**	3.910	1.485–10.29	**0.006**
% dominant side >36.4 vs ≤36.4%	1.111	0.871–1.418	0.398			
% cancer extent >57.1 vs ≤57.1%	3.207	1.288–7.984	**0.012**			
	High-risk					
	Univariate analyses			Multivariate analyses		
	HR	95%CI	*p*-value	HR	95%CI	*p*-value
PSAD >0.345 vs ≤0.345 ng/ml/cc	3.121	1.428–6.824	**0.004**	3.103	1.373–7.012	**0.006**
% positive cores >35.0 vs ≤35.0%	3.005	1.374–6.572	**0.006**			
% dominant side >40.0 vs ≤40.0	2.404	1.089–5.306	**0.030**			
% cancer extent >55.6 vs ≤55.6%	2.788	1.218–6.382	**0.015**			

*HR* Hazard ratio, *95%CI* 95% confidence interval, *% dominant side* % positive cores from dominant side, *% cancer extent* maximum % of cancer extent in each positive core, Bold indicates statistically significant

**Fig. 1:**
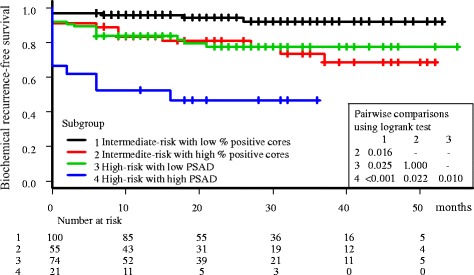
Kaplan–Meier event curves presenting biochemical recurrence-free survivals for prostate cancer patients. The entire cohort was divided into 4 subgroups by the NCCN risk, percentage of positive cores, and PSAD. Pairwise statistical comparisons among subgroups revealed that entire cohort was stratified into three distinct risk groups

Figure 1 shows the Kaplan–Meier event curves for biochemical recurrence-free survivals. The entire cohort was divided into 4 subgroups according to the NCCN risk, percentage of positive cores, and PSAD: subgroup 1 (*n* = 100): intermediate-risk with low percentage of positive cores, subgroup 2 (*n* = 55): intermediate-risk with high percentage of positive cores, subgroup 3 (*n* = 74): high-risk with low PSAD, and subgroup 4 (*n* = 21): high-risk with high PSAD. Pairwise comparisons among groups revealed that subgroup 2 and 3 showed almost the same recurrence curves, and the entire cohort could be stratified into three different risk groups. The frequency of immediate recurrence was significantly higher in subgroup 4 than in the other subgroups (33.3% versus 6.1%, *p* < 0.001).

## Discussion

Our study demonstrated that PSAD and biopsy core details had predictive value for unfavorable pathologic results and biochemical recurrence in addition to the NCCN risk classification, and there was a difference in performance characteristics for the prediction of oncologic outcomes in each NCCN risk groups. The most significant predictor for biochemical recurrence was the percentage of positive cores in the intermediate-risk group and PSAD in the high-risk PCa group. To our knowledge, this study was the first attempt to improve a stratification model of intermediate- to high-risk PCa by using the difference in performance characteristics of these preoperative factors, and the entire cohort could be stratified into three distinct risk groups. Moreover, PSAD predicted biochemical recurrence more efficiently than a NCCN sub-classification of very high-risk or high-risk, and immediate recurrence was remarkably frequent in high-risk PCa patients with high PSAD. These results suggest that both PSAD and biopsy core details are important factors for predicting the oncologic outcome of contemporary PCa patients, but those with intermediate- and high-risk PCa, which include a highly heterogeneous population, are not uniformly stratified by a single factor, and a preoperative risk classification system should consider differences in performance characteristics when incorporating these factors.

The percentage of positive cores and percentage of cancer extent in each positive core are representative factors among biopsy core details to assess cancer involvement in the prostate, and these predictive values of oncologic outcome have been investigated since the sextant biopsy era [6]. Egawa, et al. reported that these two factors in conjunction with the three known variables (PSA, clinical stage, and biopsy GS) improved the predictability of non-organ-confined PCa [15]. Meanwhile, Freeland et al. reported that a combination of PSA, biopsy GS, and percentage of cancer extent defined a preoperative model for predicting PSA recurrence [16], and almost simultaneously, they reported that the percentage of positive cores from the dominant side was a slightly better predictor of PSA recurrence than the total percentage of positive cores [17]. Briganti et al. performed a comprehensive analysis of the importance of the information contained within the variable that codes either the number or the percentage of positive cores; the percentage of cores improved stage predictions, and the number of cores improved mostly biochemical recurrence predictions [18]. The protocols of multiple core biopsy would contribute to enhance the predictive value of the biopsy core details. Briganti et al. later updated their analyses by increasing the number of cores taken from biopsy, and they repeatedly emphasized that the inclusion of the percentage of positive cores should be mandatory in the prediction model for lymph node invasion of PCa [7, 19]. Hinev, et al. demonstrated that Briganti’s nomograms showed a higher predictive accuracy for lymph node invasion as compared with the Memorial Sloan-Kettering Cancer Center nomogram, which provide predictions without information on biopsy cores [20]. Despite the positive correlation between the percentage of positive cores and the percentage of cancer extent in each positive core, each of the two factors also shows an independent predictive value for the advanced tumor stage and an increased index tumor volume in our entire cohort. Statistical superiority or inferiority between these factors might have arisen from the difference in the composition of patient data set or the saturation degree of biopsy. The relatively large number of biopsy cores might support universality as a prediction tool of these factors, but an advantage of the percentage of positive cores from the dominant side was only observed in prediction of biochemical recurrence in the entire cohort. Currently, many urologic surgeons refer to biopsy core details through prediction nomograms to assess individual risk for lymph node invasion and to make decisions regarding whether an extended resection and/or lymph node dissection should be performed during surgery.

PSAD is a convenient tool to offset the impact of prostate size that contributes to an elevated PSA and intensifies the potential value of PSA. Therefore, PSAD was initially proposed as a means of distinguishing benign hyperplasia from cancer [21]. PSAD as a predictor for adverse pathologic features or biochemical recurrence has also been debated since the middle 1990s [22]. Some studies demonstrated that PSAD provided greater advantages in predicting oncologic outcome than PSA alone in the entire cohort that underwent definitive therapy [8, 23], but conflicting results were also reported [24, 25]. Because PSAD has been incorporated into the major criteria of clinically insignificant cancer or the protocol of active surveillance for lower-risk cancer, such as Epstein or Prostate Cancer Research International: Active Surveillance (PRIAS) criteria with strict thresholds, clinical interest and discussion of PSAD have shifted toward lower risk cancers [26, 27]. Against these backgrounds, the predictive value of PSAD in risk groups of those who require definitive therapy is not more widely recognized than that of biopsy core details. Similar to our study, Koie et al. analyzed subjects limited to high-risk PCa and revealed that PSAD had independent predictive value for an adverse pathologic stage and biochemical recurrence [28]. More interestingly, Hamada et al. demonstrated that both PSAD and the percentage of cores positive from the dominant side had independent predictive value for biochemical recurrence in high-risk PCa, and they stratified patients into three groups using a statistical formula [29]. Although our study did not show the predictive value of PSAD with any threshold in intermediate-risk PCa, Narita et al. and Kang et al. demonstrated a predictive value, and Kang et al. further proposed incorporating PSAD to identify patients for whom active surveillance would be appropriate [30, 31]. The predictive value of PSAD might become conspicuous when patients are stratified into each risk group, implicitly suggesting that it is important to recognize the difference in the performance characteristics of predictive factors. Because variations in the methods for prostate size measurement may influence PSAD, such that it becomes an unstable factor, our method of estimating prostate size from MRI measurements can be recommended to minimize the fluctuation between examiners [3, 10].

Pretreatment risk stratification or nomograms must be sophisticated to make decisions for patients who need definitive therapy and for a urologic surgeon to decide upon the treatment strategy. Beyond the clinical and pathological factors, non-invasive biomarkers from urine or peripheral blood and genetic panels consisting of multiple gene profiling would help distinguish aggressive cancer from an indolent case and forecast a clinical course of PCa in a pre- and post-treatment setting [32–34]. Thus, clinical and translational research are urgently required to realize future individualized management of PCa patients. The current study has some limitations; it is a retrospective study based on a relatively small population, the median follow-up time was also short to fully determine oncological outcomes, and the results might not apply to patients of other races. The cut-off values of PSAD or biopsy core details used in this study were tentative data used to find the potential predictive impacts, and therefore, we did not intend to draw a conclusion regarding distinct thresholds for these factors. Furthermore, we did not perform lymph node dissection in all patients and the degree of dissection varies, thus we could not address the association between factors and LVI. Simultaneously, this would to a certain extent also affect the interpretation of the results of biochemical recurrence. Nevertheless, both PSAD and biopsy core details are important preoperative predictors in addition to the NCCN classification, and the findings of this study should be validated using a larger, independent dataset.

## Conclusions

Contemporary PCa patients who require definitive therapy include a highly heterogeneous population of oncological outcomes, and the established risk stratification system is insufficient to offer individualized management of PCa. PSAD and biopsy core details are important preoperative factors to predict oncologic outcomes, and should be incorporated into risk assessment for intermediate- and high-risk PCa patients. In addition, we must recognize the difference in the performance characteristics of these factors when PCa patients are stratified into each of the NCCN risk groups; where the percentage of positive cores and PSAD are independent predictors for biochemical recurrence in the intermediate- and high-risk groups, respectively.

## Additional file

Additional file 1: Table S1. Unfavorable pathological factors associated with biochemical recurrence in entire cohort. (PDF 80 kb)

## Abbreviations

GS: Gleason score
ISUP: International Society of Urological Pathology
LNI: Lymph node invasion
MRI: Magnetic resonance imaging
NCCN: National Comprehensive Cancer Network
PCa: Prostate cancer
PSA: Prostate-specific antigen
PSAD: Prostate-specific antigen density

## References

[1] Hori M, Matsuda T, Shibata A, Katanoda K, Sobue T, Nishimoto H (2015). Cancer incidence and incidence rates in Japan in 2009: a study of 32 population-based cancer registries for the Monitoring of Cancer Incidence in Japan (MCIJ) project. Jpn J Clin Oncol.

[2] Reese AC, Pierorazio PM, Han M, Partin AW (2012). Contemporary evaluation of the National Comprehensive Cancer Network prostate cancer risk classification system. Urology.

[3] Yashi M, Mizuno T, Yuki H, Masuda A, Kambara T, Betsunoh H (2014). Prostate volume and biopsy tumor length are significant predictors for classical and redefined insignificant cancer on prostatectomy specimens in Japanese men with favorable pathologic features on biopsy. BMC Urol.

[4] Koie T, Mitsuzuka K, Narita S, Yoneyama T, Kawamura S, Tsuchiya N (2015). Efficiency of pretreatment risk stratification systems for prostate cancer in a Japanese population treated with radical prostatectomy. Int J Urol.

[5] Lee SE, Kim DS, Lee WK, Park HZ, Lee CJ, Doo SH (2010). Application of the Epstein criteria for prediction of clinically insignificant prostate cancer in Korean men. BJU Int.

[6] Peller PA, Young DC, Marmaduke DP, Marsh WL, Badalament RA (1995). Sextant prostate biopsies. A histopathologic correlation with radical prostatectomy specimens. Cancer.

[7] Briganti A, Larcher A, Abdollah F, Capitanio U, Gallina A, Suardi N (2012). Updated nomogram predicting lymph node invasion in patients with prostate cancer undergoing extended pelvic lymph node dissection: the essential importance of percentage of positive cores. Eur Urol.

[8] Radwan MH, Yan Y, Luly JR, Figenshau RS, Brandes SB, Bhayani SB (2007). Prostate-specific antigen density predicts adverse pathology and increased risk of biochemical failure. Urology.

[9] NCCN Clinical Practice Guidelines in Oncology Prostate Cancer Version 2.2017-February 21, 2017. https://www.nccn.org/professionals/physician_gls/PDF/prostate.pdf Accessed 4 Jun 2017.

[10] Karademir I, Shen D, Peng Y, Liao S, Jiang Y, Yousuf A (2013). Prostate volumes derived from MRI and volume-adjusted serum prostate-specific antigen: correlation with Gleason score of prostate cancer. AJR Am J Roentgenol.

[11] Epstein JI, Allsbrook WC, Amin MB, Egevad LL, Grading Committee ISUP (2005). The 2005 International Society of Urological Pathology (ISUP) Consensus Conference on Gleason Grading of Prostatic Carcinoma. Am J Surg Pathol.

[12] Vargas HA, Hötker AM, Goldman DA, Moskowitz CS, Gondo T, Matsumoto K (2016). Updated prostate imaging reporting and data system (PIRADS v2) recommendations for the detection of clinically significant prostate cancer using multiparametric MRI: critical evaluation using whole-mount pathology as standard of reference. Eur Radiol.

[13] McNeal JE, Redwine EA, Freiha FS, Stamey TA (1988). Zonal distribution of prostatic adenocarcinoma. Correlation with histologic pattern and direction of spread. Am J Surg Pathol.

[14] Perera M, Lawrentschuk N, Bolton D, Clouston D (2014). Comparison of contemporary methods for estimating prostate tumour volume in pathological specimen. BJU Int.

[15] Egawa S, Suyama K, Matsumoto K, Satoh T, Uchida T, Kuwao S (1998). Improved predictability of extracapsular extension and seminal vesicle involvement based on clinical and biopsy findings in prostate cancer in Japanese men. Urology.

[16] Freedland SJ, Aronson WJ, Csathy GS, Kane CJ, Amling CL, Presti JC (2003). Comparison of percentage of total prostate needle biopsy tissue with cancer to percentage of cores with cancer for predicting PSA recurrence after radical prostatectomy: results from the SEARCH database. Urology.

[17] Freedland SJ, Aronson WJ, Terris MK, Kane CJ, Amling CL, Dorey F (2003). The percentage of prostate needle biopsy cores with carcinoma from the more involved side of the biopsy as a predictor of prostate specific antigen recurrence after radical prostatectomy: results from the Shared Equal Access Regional Cancer Hospital (SEARCH) database. Cancer.

[18] Briganti A, Chun FK, Hutterer GC, Gallina A, Shariat SF, Salonia A (2007). Systematic assessment of the ability of the number and percentage of positive biopsy cores to predict pathologic stage and biochemical recurrence after radical prostatectomy. Eur Urol.

[19] Briganti A, Karakiewicz PI, Chun FK, Gallina A, Salonia A, Zanni G (2007). Percentage of positive biopsy cores can improve the ability to predict lymph node invasion in patients undergoing radical prostatectomy and extended pelvic lymph node dissection. Eur Urol.

[20] Hinev AI, Anakievski D, Kolev NH, Hadjiev VI (2014). Validation of nomograms predicting lymph node involvement in patients with prostate cancer undergoing extended pelvic lymph node dissection. Urol Int.

[21] Benson MC, Whang IS, Pantuck A, Ring K, Kaplan SA, Olsson CA (1992). Prostate specific antigen density: a means of distinguishing benign prostatic hypertrophy and prostate cancer. J Urol.

[22] Seaman EK, Whang IS, Cooner W, Olsson CA, Benson MC (1994). Predictive value of prostate-specific antigen density for the presence of micrometastatic carcinoma of the prostate. Urology.

[23] Freedland SJ, Wieder JA, Jack GS, Dorey F, deKernion JB, Aronson WJ (2002). Improved risk stratification for biochemical recurrence after radical prostatectomy using a novel risk group system based on prostate specific antigen density and biopsy Gleason score. J Urol.

[24] Ingenito AC, Ennis RD, Hsu IC, Begg MD, Benson MC, Schiff PB (1997). Re-examining the role of prostate-specific antigen density in predicting outcome for clinically localized prostate cancer. Urology.

[25] Brassell SA, Kao TC, Sun L, Moul JW (2005). Prostate-specific antigen versus prostate-specific antigen density as predictor of tumor volume, margin status, pathologic stage, and biochemical recurrence of prostate cancer. Urology.

[26] Bastian PJ, Mangold LA, Epstein JI, Partin AW (2004). Characteristics of insignificant clinical T1c prostate tumors. A contemporary analysis. Cancer.

[27] Bul M, Zhu X, Valdagni R, Pickles T, Kakehi Y, Rannikko A (2013). Active surveillance for low-risk prostate cancer worldwide: the PRIAS study. Eur Urol.

[28] Koie T, Mitsuzuka K, Yoneyama T, Narita S, Kawamura S, Kaiho Y (2015). Prostate-specific antigen density predicts extracapsular extension and increased risk of biochemical recurrence in patients with high-risk prostate cancer who underwent radical prostatectomy. Int J Clin Oncol.

[29] Hamada R, Nakashima J, Ohori M, Ohno Y, Komori O, Yoshioka K (2016). Preoperative predictive factors and further risk stratification of biochemical recurrence in clinically localized high-risk prostate cancer. Int J Clin Oncol.

[30] Narita S, Mitsuzuka K, Tsuchiya N, Koie T, Kawamura S, Ohyama C (2015). Reassessment of the risk factors for biochemical recurrence in D’Amico intermediate-risk prostate cancer treated using radical prostatectomy. Int J Urol.

[31] Kang HW, Jung HD, Lee JY, Kwon JK, Jeh SU, Cho KS (2016). Prostate-specific antigen density predicts favorable pathology and biochemical recurrence in patients with intermediate-risk prostate cancer. Asian J Androl.

[32] Sharma P, Zargar-Shoshtari K, Pow-Sang JM (2015). Biomarkers for prostate cancer: present challenges and future opportunities. Future Sci OA.

[33] Behesnilian AS, Reiter RE (2015). Risk stratification of prostate cancer in the modern era. Curr Opin Urol.

[34] Ross AE, Johnson MH, Yousefi K, Davicioni E, Netto GJ, Marchionni L (2016). Tissue-based Genomics Augments Post-prostatectomy Risk Stratification in a Natural History Cohort of Intermediate- and High-Risk Men. Eur Urol.

