# Supplementary material for: Performance characteristics of prostate-specific antigen density and biopsy core details to predict oncological outcome in patients with intermediate to high-risk prostate cancer underwent robot-assisted radical prostatectomy
Source: BMC Urol. 2017 Jun 23;17:47. doi: 10.1186/s12894-017-0238-y (PMC5481958; doi:10.1186/s12894-017-0238-y)
Supplement: Additional file 1: Table S1. — Unfavorable pathological factors associated with biochemical recurrence in entire cohort. (PDF 80 kb) [file 12894_2017_238_MOESM1_ESM.pdf]

Table S1 Unfavorable pathological factors associated with biochemical recurrence in entire cohort

	Univariate analyses			Multivariate analyses		
	**HR**	**95%CI**	***p*-value**	**HR**	**95%CI**	***p*-value**
Tumor stage ≥pT3 vs ≤pT2	5.098	2.787-9.324	**<0.001**	3.501	1.876-6.535	**<0.001**
Index tumor volume >0.718 vs ≤0.718 cc	4.984	1.966-12.63	**<0.001**	3.411	1.317-8.833	**0.012**
Gleason score ≥8 vs ≤7	3.071	1.692-5.574	**<0.001**	2.494	1.358-4.583	**0.003**
Surgical margin status positive vs negative	3.815	2.099-6.936	**<0.001**			

HR Hazard ratio, *95%CI* 95% confidence interval, Bold indicates statistically significant.
